# Adolescent Perceptions of an Online Safety Chatbot: Survey Study

**DOI:** 10.2196/71498

**Published:** 2026-02-12

**Authors:** Meriel Charles, James D Sauer, Erin Roehrer, Jeremy Prichard, Paul Watters, Joel Scanlan

**Affiliations:** 1 School of Psychological Sciences University of Tasmania Hobart Australia; 2 School of Information and Communication Technology University of Tasmania Hobart Australia; 3 School of Law University of Tasmania Hobart Australia; 4 Cyberstronomy Melbourne Australia

**Keywords:** adolescent harms, adolescent, artificial intelligence, chatbot, mental health services, online safety, trust, user-centered design

## Abstract

**Background:**

Adolescents face a variety of potential harms in the online environment, including exposure to distressing illegal material, cyberbullying, image-based abuse, and “sextortion.” Various agencies provide on-demand helpline and information services for children and adolescents to support them with navigating online (and offline) harms.

**Objective:**

This study examined whether a chat-based conversational agent (chatbot) might be a useful additional tool for meeting the needs of adolescents at risk from online harms. We developed a prototype chatbot—including both conversational and menu-driven user options—and evaluated users’ trust in the chatbot. In this context, trust relates to perceptions of the chatbot’s usability and the value of the information and support it provides.

**Methods:**

Participants (n=224; mean age 16.8 years) interacted with the chatbots and evaluated them in terms of user trust: perceived usability and utility (ie, relevance of support resources provided).

**Results:**

Most participants (conversational chatbot: 141/224, 63% and menu-driven chatbot: 142/224, 63%) showed a willingness to click on the chatbots’ recommended support links. Participants with higher trust in the chatbots were more likely to click the links for recommended support services (with extreme evidence for large effects: δ=0.73, 95% credible interval [CrI] 0.46-1.00 and δ=0.78, 95% CrI 0.50-1.07, for the conversational and menu-driven chatbots, respectively; Bayes factor [BF_10_]>50,000), and participants who clicked the links, compared with those who did not, reported higher rates of positive attitudes toward their decision (with extreme evidence for large effects: δ=0.87, 95% CrI 0.58-1.15 and δ=0.84, 95% CrI 0.54-1.12, for the conversational and menu-driven chatbots, respectively; BF_10_>3,000,000). The conversational and menu-driven chatbots differed little in perceived trust or effectiveness.

**Conclusions:**

Chatbots represent a promising additional tool to help adolescents access mental health–related support services and navigate online harms. However, establishing trust is critical.

## Introduction

### Overview

Chat-based conversational agents—chatbots—are used in many contexts globally. Research on technology in the health care and service sector has found that the use of chatbots (compared to traditional systems, such as web interfaces or menu-based systems) can lead to a more positive user experience (ie, users feeling more comfortable and better understood) [1-4]. This is often attributed to chatbots facilitating a more conversational and interactive form of communication, making users feel more engaged and better supported [4]. Developing these feelings of engagement and support is essential. In health care and mental health support contexts, individuals often need to share personal information with a chatbot (eg, their medical history or symptoms) to receive customized recommendations [5-8]. This process relies on users feeling secure in sharing sensitive information [2,9-12]. Effectively, the successful adoption of human-technology systems (like chatbots) relies on people’s trust in these systems [3,6,13-19]. Trust in human interactions is grounded in shared emotional connections and the confidence a person has that others will prioritize their interests [19,20]. However, trust in technology and artificial intelligence (AI) systems operates differently due to the absence of genuine empathy [11,19,21]. Instead, trust in human-technology interactions is more transactional, being influenced by factors such as functionality, performance, helpfulness, predictability, and reliability [13,19]. Specifically, trust in conversational chatbots has been defined as the users’ willingness to provide confidential information and to accept and act on the chatbot’s recommendations [17]. In this study, we tested adolescents’ feelings of trust toward, and perceptions of the utility of, 2 chatbot systems, providing proof-of-concept evidence for the potential utility of these chatbots in providing support to adolescents at risk from online harms.

Developing trust involves both cognitive and affective processes. To facilitate cognitive trust, a system must meet expectations and be perceived as useful. In contrast, affective trust is related to emotional connections, such as making a user feel secure and comfortable [22,23]. Cognitive trust, grounded in reasoning, is associated with practical aspects of an information system (ie, usage-related factors), such as functionality, ease of use, and the quality and relevance of its recommendations [16]. When users consider using new technology-based services, they can engage in a rational decision-making process, weighing potential benefits against drawbacks [22,24]. Building and reinforcing cognitive trust occurs when users experience tangible, positive outcomes (eg, when they perceive that the new system makes tasks easier). When users feel their needs are met, they are more likely to trust the system and continue using it [25-28]. Cognitive trust positively influences the acceptance and usage of technology; conversely, technology with low cognitive trust is less likely to be adopted [22,29]. Thus, developing an online safety chatbot that meets users’ needs is essential for establishing and sustaining cognitive trust and for the ultimate success of the system.

Affective trust is not based solely on objective measures of performance but rather on feelings associated with security, comfort, and satisfaction [22,30]. Affective trust can help reduce the psychological distance, or perceived emotional gap, between a user and the information system [3,30-32]. Compared to basic information systems (such as a web or menu-style interface), chatbots, with their more natural interaction and personalized conversational approach, can reduce psychological distance by making users feel more comfortable and supported, encouraging users to share personal information and accept support recommendations from the chatbot [3,17,33,34]. Even adding a basic human-shaped symbol, compared to a machine-like symbol, to a chatbot has been found to enhance affective trust, reduce psychological distance between the user and the chatbot, and increase users’ intentions to comply with chatbot recommendations [3].

Cognitive and affective trust often reinforce each other [22,35,36]. When a person believes a technology is useful, they become emotionally engaged with it. When they are emotionally engaged, they are more likely to view the system as useful and credible. Importantly, when users trust a chatbot, they are more likely to follow its recommendations, making trust a critical factor in predicting users’ behavioral intentions, their willingness and motivation to follow advice [3,17,37-39]. A person’s behavioral intention serves as an informative, though admittedly imperfect, predictor for actual behavior [40-43]. Several studies show a positive link between a stated positive intention to use an AI service (such as a chatbot) and actual usage [26,44]. Thus, investigating trust-related factors and how they relate to potential help-seeking behavior in the context of an online safety chatbot is essential for predicting how users might perceive and interact with this potentially helpful tool, as well as their willingness to accept and act on its recommendations, in a real-world setting.

### The Case for an Online Safety Chatbot for Adolescents

Adolescents encounter many challenges staying safe online. The online and social media environments present a range of risks, including cyberbullying and exposure to inappropriate content, which can have serious consequences for adolescent well-being [45]. The pressures of negative online interactions can contribute to increased levels of stress, anxiety, and depression among adolescents [46,47]. The “Digital Lives of Aussie Teens” report by the e-Safety Commissioner [45] found that adolescents in Australia spend, on average, 14 hours online each week (researching topics of interest and engaging with social media, entertainment, and gaming) and, among those aged 14-17 years, just over 50% reported negative experiences in the past 6 months. Such incidents include being contacted by strangers, receiving threats and abuse, and being exposed to inappropriate or unwanted content. In addition, one-third reported negative online experiences stemming from instances of bullying that occurred at school. Although many adolescents do seek help from friends and family, a considerable portion do not share their concerns or report harmful incidents to authorities (eg, law enforcement and the school), the online platform (eg, social media platform and gaming websites), or support services (eg, helplines, mental health professionals, and counsellors). Nonetheless, a large majority (75%) of adolescents expressed a need for online safety information, with schools and reputable websites for digital well-being being their preferred sources for accessing this information. Importantly, an online safety chatbot can serve a dual purpose, not only supporting those who may have experienced harm but also reducing the risk that they may unintentionally be causing harm to others due to their limited understanding of legalities, consent, and privacy rights. By providing guidance and information on these topics, the chatbot can contribute to the promotion of responsible online behavior among adolescents. The vulnerability of adolescents to online threats, and the potentially harmful impact of such experiences on their development and mental health, highlight the need to address cyber safety concerns among youth and ensure they have easy access to reliable information from trusted sources.

Yourtown [48], which operates Commonwealth-funded services for children across Australia, has reported that the use of on-demand helplines and websites by minors has steadily increased between 2018 and 2023. This paper suggests that chatbots could be a useful additional tool for helping address cyber safety and related mental health concerns among adolescents [49]. Integrating an online safety chatbot service into a school program could not only raise awareness of the risks associated with negative online interactions but also serve as an important resource for students seeking help. This online safety chatbot can provide direct links to information tailored to the students’ concerns on issues such as cyberbullying, grooming, image-based abuse, and “sextortion,” online gambling, eating disorders, and substance abuse—as well as counselling services, mental health resources, and trusted helplines. This tool could be particularly important in reaching those who may feel embarrassed or hesitant when talking to others, or who lack adequate support from family members or peers, leaving them without necessary guidance on these issues. An online safety chatbot service available on school websites could help schools bridge this gap by providing a readily accessible platform where students can seek help anonymously. By offering an additional avenue to access trustworthy information, an online safety chatbot can empower vulnerable adolescents to address their concerns proactively and confidentially and direct students to appropriate mental health support. It can also complement school and community efforts to promote online safety and well-being among young people.

Given the sensitive nature of online safety concerns, especially where adolescents may be coping with distressing emotions, a chatbot service can provide a safe and confidential space for adolescents to share their concerns [31]. This would allow the online safety chatbot to offer tailored recommendations that can be delivered empathetically and securely to help young people navigate the issues they are experiencing. This would be particularly helpful for those who may feel more comfortable asking about sensitive issues or sharing personal information to a dedicated online safety platform rather than a human [50]. However, as noted previously, the efficacy of a chatbot service is likely to be associated with users’ trust in the chatbot, which, in turn, will affect how receptive users are to the chatbot’s recommendations for further support.

### This Study

In technology design, creating user-friendly systems is essential, particularly in sensitive information-sharing contexts [2,3,6]. When implementing an online safety chatbot in schools to help address online harms, it is important to include aspects of cognitive trust (eg, perceived utility) and affective trust (eg, feeling comfortable with its use). Several trust-related factors can influence how likely someone is to adopt and engage with a chatbot [3,16-19,26,51]. These include perceptions of the accuracy of the information the chatbot provides, how easy and user-friendly the chatbot is to interact with, and how well its suggestions align with a user’s needs. The chatbot’s ability to provide relevant recommendations based on the user’s input, such as offering resources related to their specific concern, is important to building and maintaining trust. Additionally, feelings of trustworthiness, security, and positivity during interactions with the chatbot play a key role in impacting users’ overall perception and willingness to continue using it [2,3,10-12,52]. Essentially, a user’s perception of the chatbot’s reliability and the more helpful and comfortable they feel with the chatbot, the more likely they may be to trust it and follow its recommendations for additional help. In this study, we included 6 measures related to trust in chatbots, tapping both cognitive and affective trust (accuracy, user-friendliness, relevant recommendations, trustworthiness, security, and positivity).

### Menu-Driven and Simple Conversational Chatbot

To examine the potential for developing an online safety chatbot for a school or community setting, participants interacted with both a conversational and menu-based chatbot using a scenario-based activity (as explained further in the Methods section). A menu-driven chatbot offers users preselected options, guiding users through structured pathways based on their selections. In contrast, a conversational chatbot enables users to input questions or concerns using natural language. There are 2 main types of conversational chatbots: “simple chatbots” and “smart chatbots.” Smart chatbots use advanced algorithms and AI to understand and respond to user inputs in a more flexible and natural manner. In contrast, a simple conversational chatbot, the system used in this study, simulates simple conversations, providing tailored responses and recommendations based on user textual input. They leverage advanced machine learning models to understand user input (trained on input phrases and rules) to establish user intent but then rely on predefined responses to provide answers to user input. Phrases like “Sorry, I did not understand” appear for inputs that do not match preselected intents, particularly in contexts where a less constrained chatbot may respond with unhelpful or inappropriate responses. There have been many examples in recent years of chatbots responding poorly, although none more famously than @TayAndYou [53]. The choice to implement a simple chatbot in this study was to minimize the risk to adolescents inquiring about sensitive topics.

Examining users’ ratings for trust-related factors across the 2 chatbots allowed us to compare which method of interaction and design approach might better align with students’ needs and preferences when seeking help navigating online safety issues. This study, being exploratory and intended as a proof of concept, investigates multiple research questions and considers alternative viewpoints. For example, students might trust a menu-driven chatbot more because it is predictable and easy to navigate with clear menu options, but this might feel less interactive and personalized compared to a conversational chatbot [54]. However, a simple conversational chatbot, though potentially more flexible and engaging, may be limited when handling complex sentences or questions. This could affect the accuracy of its responses, potentially impacting trust in the chatbot. A preference for a menu-driven chatbot or a conversational chatbot may depend on whether students prioritize a structured interface or a more conversational interaction.

Past research on technology adoption suggests perceptions of an online safety chatbot’s utility may be influenced by gender-specific factors [16], highlighting the importance of considering these differences. For example, findings suggest that females tend to have a favorable view if they perceive the technology as practical, while males might need extra motivation to engage [55,56]. Thus, we examined potential gender differences in students’ trust and their engagement with the chatbot’s recommendations, as this may inform an online safety chatbot’s marketing and design to better cater to the preferences of different gender groups, enhancing its effectiveness.

In sum, this study examined whether a chatbot might be a useful additional tool for meeting the needs of adolescents at risk from online harms. Specifically, in this proof-of-concept work, we evaluated users’ (1) perceptions of efficacy for 2 types of chatbot, (2) trust in our chatbots, and (3) preferences for chatbot design features.

### Research Questions

Textbox 1 outlines the study’s research questions, which examine students’ help-seeking behavior and their perceptions of trust and usability in chatbot systems. The questions focus on the relationships among key trust factors, behavioral intention to use chatbots, and differences between conversational and menu-driven designs. Additional areas of inquiry include user preference, personalization, anonymity, prior experience, gender differences, and common usability challenges, providing an overall framework for understanding trust and user experience in chatbot-based support.

**Research questions examining students’ trust, usability perceptions, and behavioral intentions toward conversational and menu-driven chatbot systems.**

**Research questions**

- How likely are students to seek help from outside their family and peer group?
- Do perceived accuracy, usefulness, ease of use, security, comfort, and credibility show a positive correlation, indicating that they may work together to contribute to trust in chatbot systems? Does this differ between conversational and menu-driven chatbots?
- Is there a relationship between a higher trust score (combined scores on perceived usefulness, ease of use, security, comfort, and credibility) and behavioral intention to use a chatbot (measured by clicking on a help link and positive feelings about the action)? Does this differ between conversational and menu-driven chatbots?
- Do conversational chatbots have a higher overall trust score than menu-driven chatbots, indicating that they are perceived as more trustworthy (based on perceived usefulness, ease of use, security, comfort, and credibility ratings)?
- Does perceived usefulness differ between conversational and menu-driven chatbots?
- In terms of perceived ease of use, is there a preference for conversational chatbots over menu-driven chatbots?
- Overall, do users prefer a conversational or menu-driven chatbot, and what factors influence users’ preferences? (Qualitative)
- Is there a preference for personalization of the conversational chatbot? Is a face or name important?
- Is there a preference for strict anonymity in a chatbot system?
- How does prior experience with conversational chatbots affect users’ perceptions of ease of use, comfort, and overall trust (trust score) in a conversational chatbot?
- What are the common difficulties encountered by users when using conversational and menu-driven chatbots, and how might these difficulties relate to user experience (“ease of use”) and trust in the system (“trust score”)? (Qualitative)
- Do trust scores differ according to user gender identity? Does gender impact a user’s behavioral intention?

## Methods

### Ethical Considerations

All procedures were compliant with the relevant laws and institutional guidelines and have been approved by the appropriate institutional committee (March 26, 2024; project no: H0029893). Privacy rights for human subjects were observed, and informed consent was obtained prior to data collection. All data were collected anonymously and stored securely in line with the university research data governance guidelines. Participants completed the survey within their “home room” group at school, within the presence of a school staff member. A member of the research team was also present to answer questions from participants. The information sheet also provided guidance on contacting the school counselling team or 1 of 2 listed independent support lines (Beyond Blue and Headspace) if the survey caused any discomfort or raised concerns for participants.

### Participants

A total of 224 participants (131 female, 93 male, and 0 selecting a different identification), with a mean age of 16.8 years, completed the study. Participants were recruited from a local school, and both participant and parent or guardian information sheets were prepared and communicated to the relevant parties through the school communication system. This same system also provided a facility for parents or guardians to provide consent for participants. The school collected the list of students for whom consent was provided, and this list was later used to provide access to the survey to students who met the consent requirements.

### Materials

**Figure 1:**
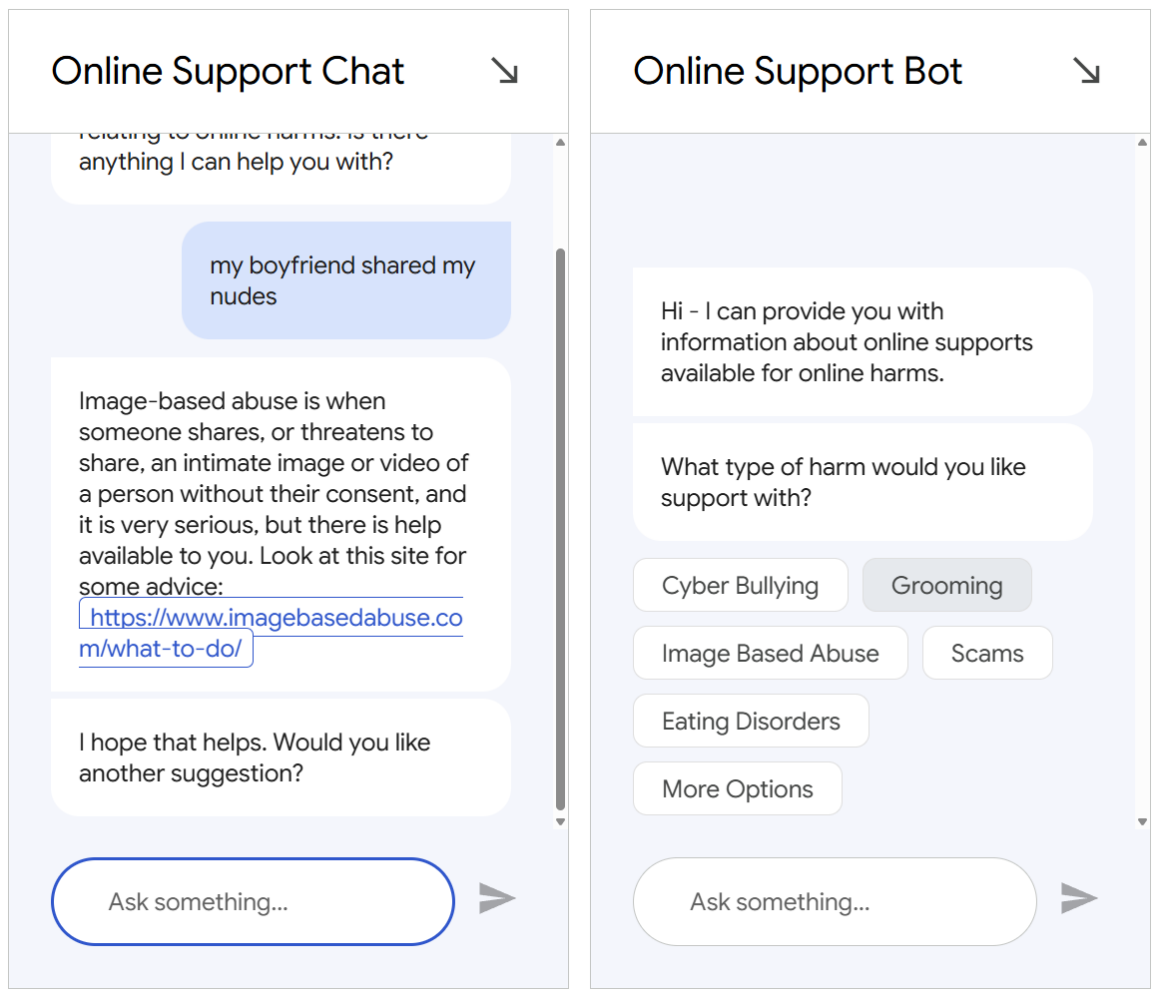
The 2 variants of the chatbot created within the project: the conversational chatbot (left) and menu-driven chatbot (right). The conversational chatbot allows the user to enter free text and engage in a conversation. The menu-driven chatbot prompts users to select response from prespecified lists.

The chatbot was developed using a co-design process, leveraging 2 focus groups at a local school that teaches grades 11 and 12. The first focus group comprised members of the pastoral care team at a school, and the second was a cohort of students. This design process is detailed in Roehrer et al [57]. The focus groups considered what harms were of greatest importance to be included, what resources were of most value, and general design considerations, including what the chatbot’s opening line to users should be. The chatbot design did not attempt to mimic a young person’s vocabulary or style in alignment with similarity-attraction theory [58] but aimed to use simple casual language that would be understandable at a 13-year-old reading level. The focus groups highlighted the importance of nonjudgmental advice that was factual and trustworthy. The chatbot included slang and other terms used by young people within its training data to understand inputs, but its responses did not include such terms and prioritized simple plain English responses that directed users to support resources and services. The chatbot was built on the Google Dialogflow platform [59] based on the outputs of the design requirements and in 2 variants, 1 menu-driven and 1 conversational (Figure 1). The survey was delivered to participants using the Qualtrics platform, accessed through a web link provided to them in the classroom at the time of the survey. The chatbot variants were embedded within the survey, with a randomized order of presentation of the 2 chatbot types. Following the survey, lunch was provided on the school campus for students who participated in the survey.

### Procedure

After reading the task instructions, participants were asked to enter their gender and age into text boxes. In addition, they were asked a single question about their general help-seeking behavior, “When something worries you, how likely are you to seek help from sources outside of your family or peer group?” (7-point scale: “very unlikely” to “very likely”). The main task consisted of 2 parts. In the first part of the task, participants engaged with the first chatbot (eg, conversational chatbot). They began by reading the first vignette, which provided context for the interaction with the chatbot. Participants entered into a simulated “conversation” with the first interactive chatbot, assuming the role of the character in the given scenario. Based on the details shared by the participant, relevant hyperlinks to appropriate websites, such as eSafety [60] and the Butterfly Foundation [61], were embedded within the “conversations.” These interactive clickable links led directly to real-world websites. For example, if the conversation related to cyberbullying, participants received a hyperlink directing them to eSafety. Additionally, the chatbot offered the option for another link to a different but relevant website. Participants received instructions at the start of the study that these hyperlinks were interactive. After engaging with the first vignette, participants proceeded to a second vignette, repeating the process (using the same interactive chatbot). After completing both interactions, participants responded to a set of 8 questions aimed at capturing their thoughts related to their experiences with the first chatbot. The following questions were asked for conversational and menu-driven chatbots following an interaction with that system and were aimed at capturing behavioral intention. Question 1: “For either vignette, did your character choose to click a support link?” (This was aimed at capturing behavioral intention). Question 2: “How did your character feel about this decision? e.g., did they feel positive about their decision, or did they have some reservation?” The participants then answered 6 questions on various aspects of trust-related factors. Participants were instructed to consider the perspective of the person seeking help while responding to these questions. The following questions related to trust with responses collected on a 5-point Likert scale, ranging from “strongly disagree” to “strongly agree”: (1) “The interaction with the chatbot resulted in a recommendation for a relevant service” (perceived usefulness), (2) “Overall, I felt the chatbot provided accurate information” (perceived accuracy), (3) “The interaction with the chatbot felt user-friendly” (perceived ease of use), (4) “The interaction with the chatbot felt secure in terms of privacy” (perceived security), (5) “I felt that the interaction with the chatbot was a positive experience” (perceived comfort), and (6) “I found the chatbot to be trustworthy” (perceived credibility). Higher scores indicate an increased likelihood of trust in the chatbot. In the second part of the task, participants engaged with a different chatbot (eg, menu-driven chatbot). As in the first part, participants read 2 new vignettes, and engaged in a “conversation.” Participants then answered the same set of 8 questions as in the first part. This approach allowed us to collect information on participants’ engagement with the 2 distinct interactive chatbots (conversational and menu-driven), their perceptions of those interactions, and the likelihood of seeking further advice through a suggested helpline.

At the end, participants were presented with a final set of questions designed to gather their feedback and impressions directly comparing the 2 chatbot systems: Question 1: “After engaging with both systems, which did you find most helpful?” (Response options: conversational, menu-driven). Question 2: “After engaging with both systems, which did you find easier to use?” (Response options: conversational, menu-driven). Question 3: “If you wanted to find out more about support services and information related to online harms, which system would you prefer to use?” (Response options: conversational, menu-driven, neither). Question 4: “Do you believe the conversational chatbot should be personalised, for example, having a face or a name?” (Response options: yes, no, unsure). Question 5: “Do you think a chatbot service for information related to online harms should be strictly anonymous?” (Response options: yes, no, unsure). Question 6: “Before today, have you used a conversational chatbot before?” (Response options: yes, no, unsure). Question 7: “Please share your thoughts on what specific features would increase your comfort level when it comes to sharing personal information with a chatbot.” Question 8: “Did you encounter any difficulties using either of the systems today?” For Questions 3, 4, 5, 7, and 8, participants were provided a text box to add any additional information.

### Analysis Strategy

We used the *jamovi* [62] packages to run Bayesian analyses to examine our data. This approach provides 2 key advantages for our purposes. First, unlike null hypothesis significance testing (NHST), Bayesian analyses can quantify evidence for the null hypothesis. When a NHST approach returns a nonsignificant result, all that can be concluded is that there was no evidence of a meaningful difference between groups or association between variables. In contrast, a Bayesian approach allows researchers to draw meaningful conclusions about equivalence across conditions or the absence of associations between variables. Second, a Bayesian approach allows for targeted *t* tests and chi-square analyses without inflating the risk of Type I error [63]. When interpreting Bayes factors (BFs), we relied on Lee and Wagenmakers’ [64] criteria, where evidence for the alternative (or null) hypothesis can be categorized as follows: 1-3=anecdotal, 3-10=moderate, 10-30=strong, 30-100=very strong, and >100=extreme. For Bayesian *t* tests, effect size measures are reported as δ, the population equivalent to Cohen *d* [65]. An adapted summative content analysis was performed on the survey responses, using the comments to drive the units of meaning, providing labels and then grouping labels into a final category [66]. Our analyses were largely exploratory, but the preregistration for our analysis strategy can be found here at the Open Science Framework [67].

## Results

### Overview

A key aim of this study was to examine aspects of trust in both a conversational and menu-driven online safety chatbot. As individuals typically require trust before they comply with recommendations [11,21], establishing and maintaining trust is an essential component to ensuring adolescents are receptive to its suggestions for related online e-safety resources.

### Willingness to Seek Help From External Sources

Establishing whether young people are open to seeking help beyond their close friends and family circle is important, as this informs the need for and development of effective external support strategies. When participants were asked about the likelihood of seeking help outside their family and peer group, of the 224 participants, 39% (87/224) reported it was likely (collapsed across slightly, moderately, and very likely), 42% (94/224) said it was unlikely (collapsed across slightly, moderately, and very unlikely), with the rest (n=43) being unsure. Thus, over one-third of students showed openness to seeking external help when needed. We measured students’ willingness to follow a chatbot’s recommended service by including clickable hyperlinks in their chatbot interactions and asking participants about their link-clicking behavior. We considered those who clicked on a help link might also be more likely to seek help when presented with that opportunity in real-life. When engaging with both the conversational and menu-driven chatbots, a substantial majority of the 224 participants reported they clicked on a help link (141/224, 63% and 142/224, 63% for the conversational and menu-driven chatbots, respectively). As students were no more or less likely to press on a help link via a conversational or menu-driven chatbot, this suggests that the type of interface (as they currently stand) does not influence help-seeking behavior. Although females were, numerically, more likely than males to click on help links provided by both the menu-driven (90/131, 69% vs 52/93, 56%) and conversational chatbots (87/131, 66% vs 54/93, 58%), the evidence in favor of these representing meaningful differences was anecdotal (BF_10_<2.10).

Considering an individual’s attitude, whether positive or negative, toward a behavior is important, as it can indicate the likelihood of engaging in that behavior in the future [42]. Positive feelings associated with a behavior often suggest a higher chance of repeating that behavior. Therefore, a positive feeling toward clicking a link in this study might suggest a tendency to seek assistance via a chatbot-suggested link in real-world situations. Bayesian independent samples 2-tailed *t* tests found that, for both the conversational (BF_10_=1.29×10^7^) and menu-driven chatbots (BF_10_=3.73×10^6^), there was extreme evidence that students who clicked on the provided link, compared with those who did not, felt more positive about their choice. For both the conversational (mean 3.50, SD 0.78 vs mean 2.76, SD 0.92; δ=0.87, 95% credible interval [CrI] 0.58-1.15) and the menu-driven chatbots (mean 3.49, SD 0.85 vs mean 2.82, SD 0.63; δ=0.84, 95% CrI 0.54-1.12), these differences exceeded the threshold for large effects. These findings suggest that choosing to click on a link while interacting with either the conversational or menu-driven chatbot is associated with a higher level of positivity regarding that decision, in contrast to choosing not to seek help via the link.

### Trust and Trust-Related Factors

Trust is an important factor in users’ willingness and motivation to follow advice (ie, behavioral intentions; [6,17,26]. Thus, we were interested in how trust relates to clicking on chatbot-suggested help links. We expected that higher overall trust in the chatbot should relate to a greater likelihood of clicking on a suggested help link. A Bayesian independent samples 2-tailed *t* test (1-tailed/2-tailed) showed extreme evidence (BF_10_=87,421) for a large effect (δ=0.73, 95% CrI 0.46-1.00) such that, for the conversational chatbot, those who clicked on a help link (mean 3.68, SD 0.75) had a higher trust score (using the mean of the 6 trust-related factors) than those who did not click (mean 3.14, SD 0.65). Similarly, for the menu-driven chatbot, there was extreme evidence (BF_10_=543,051) for a large effect (δ=0.78, 95% CrI 0.50-1.07), where those who clicked on a help link (mean 3.76, SD 0.73) had a higher trust score compared with those who did not (mean 3.22, SD 0.53). These findings are consistent with the idea that trust relates to a person’s inclination to use the chatbot’s suggested help features.

We compared participants’ perceptions of menu-driven and conversational chatbots across various trust-related factors. As trust is a multifaceted concept, it is important to first consider the relationship between these factors and their potential collective impact on trust in chatbots. Key trust-related factors influencing chatbot adoption, which are essential for developing and maintaining trust, include the accuracy of information based on user input, user-friendliness, appropriateness of recommendations (eg, directing users to relevant help sites for specific concerns such as cyberbullying), and feelings of trustworthiness, security, and positivity toward interactions with the chatbot. The internal consistency of the trust-related factors in our study was high, with a Cronbach α coefficient of 0.88, indicating good reliability among the measured items (Tables 1 and 2). We found extreme evidence for moderate-to-strong correlations between all trust-related factors for both menu-driven and conversational chatbots, highlighting the factors’ interconnectedness.

**Table 1:** Bayesian correlation matrix showing association between trust-related factors for the menu-driven chatbot.

Variable		Accuracy	User-friendly	Secure	Trustworthy	Positiveness	Recommended relevant
**Accuracy**							
	*r*	—^a^					
	BF_10_	—					
**User-friendly**							
	*R*	0.556	—				
	BF_10_	3.64 × 10^16^	—				
**Secure**							
	*R*	0.476	0.442	—			
	BF_10_	1.60 × 10^11^	1.94 × 10^9^	—			
**Trustworthy**							
	*R*	0.545	0.498	0.714	—		
	BF_10_	5.85 × 10^15^	3.66 × 10^12^	1.13 × 10^33^	—		
**Positiveness**							
	*R*	0.577	0.674	0.474	0.564	—	
	BF_10_	1.85 × 10^18^	8.15 × 10^27^	1.27 × 10^11^	1.70 × 10^17^	—	
**Recommended relevant**							
	*R*	0.598	0.482	0.465	0.571	0.519	—
	BF_10_	1.08 × 10^20^	3.39 × 10^11^	3.53 × 10^10^	6.23 × 10^17^	8.33 × 10^13^	—

^a^Not applicable.

**Table 2:** Bayesian correlation matrix showing association between trust-related factors for the conversational chatbot.

Variable		Accuracy	User-friendly	Secure	Trustworthy	Positiveness	Recommended relevant
**Accuracy**							
	*R*	—^a^					
	BF_10_	—					
**User-friendly**							
	*R*	0.636	—				
	BF_10_	5.42 × 10^23^	—				
**Secure**							
	*R*	0.398	0.515	—			
	BF_10_	1.42 × 10^7^	4.57 × 10^13^	—			
**Trustworthy**							
	*R*	0.514	0.578	0.704	—		
	BF_10_	3.84 × 10^13^	2.07 × 10^18^	4.48 × 10^31^	—		
**Positiveness**							
	*R*	0.668	0.681	0.600	0.631	—	
	BF_10_	1.35 × 10^27^	4.82 × 10^28^	1.93 × 10^20^	1.61 × 10^23^	—	
**Recommended relevant**							
	*R*	0.550	0.501	0.400	0.422	0.483	—
	BF_10_	1.30 × 10^16^	5.23 × 10^12^	1.79 × 10^7^	2.00 × 10^8^	4.15 × 10^11^	—

^a^Not applicable.

Table 3 displays the means and SDs for, and Bayesian 2-tailed paired samples *t* tests comparing, each trust-related factor across chatbot types. Across these comparisons, 3 key findings emerge. First, for most trust-related factors, there is evidence of equivalence across chatbot types, indicating that users did not report trusting one chatbot over the other. This evidence was strong for perceptions of security and trustworthiness, moderate for users’ feelings of positiveness toward the chatbots and for the perceived relevance of the recommended information, but only anecdotal for perceptions of the accuracy of provided information. Second, there was moderate evidence that users viewed the menu-driven chatbot as more user friendly than the conversational chatbot, though the effect size only approach the cutoff to be considered small. Finally, there was anecdotal evidence suggesting that overall trust scores may have favored the menu-driven chatbot over the conversational chatbot. However, this interpretation requires 2 caveats: the effect size here was trivial, and any difference likely reflects differences in perceived user-friendliness. Together, these findings suggest that users generally reported equivalent trust for conversational and menu-driven chatbots, but that the conversational chatbot may need to be refined to improve usability.

**Table 3:** Mean (SD) for, and Bayesian paired samples 2-tailed t tests comparing, trust scores for the menu-driven and conversational chatbots.

Variable	Menu-driven chatbot, mean (SD)	Conversational chatbot, mean (SD)	BF_10_^a^	δ (95% CrI^b^)
Accuracy	3.54 (0.93)	3.42 (0.96)	2.46^a^	0.12 (–0.01 to 0.25)
User-friendly	3.62 (0.91)	3.45 (1.00)	3.09	0.18 (0.05 to 0.31)
Secure	3.51 (0.89)	3.48 (0.93)	10.65^a^	0.04 (–0.09 to 0.17)
Trustworthy	3.50 (0.91)	3.50 (0.94)	13.18^a^	0.01 (–0.12 to 0.14)
Positiveness	3.51 (0.88)	3.44 (0.93)	6.42^a^	0.08 (–0.05 to 0.21)
Recommended relevant	3.70 (0.90)	3.59 (1.02)	3.49^a^	0.11 (–0.02 to 0.24)
Overall trust	3.56 (0.71)	3.48 (0.76)	1.45	0.16 (0.03 to 0.29)

^a^Indicates Bayes factor (BF_01_) expressing evidence in favor of the null/equivalence across chatbot types.

^b^CrI: credible interval (around the effect size).

When examining gender effects on overall trust, Bayesian independent samples *t* tests revealed anecdotal (BF_01_=2.40; δ=0.19, 95% CrI –0.07 to 0.45) and moderate evidence for the null (BF_01_=5.12; δ=0.09, 95% CrI –0.17 to 0.36) for the menu-driven and conversational chatbots, respectively. Thus, for both types of chatbot, female and male users reported similar levels of trust (mean 3.62, SD 0.63 and mean 3.48, SD 0.82, for female and male users rating the menu-driven chatbot; and mean 3.51, SD 0.70 and mean 3.43, SD 0.84, for female and male users rating the conversational chatbot).

### User Preferences

Despite evidence of greater user-friendliness when considering the trust-related factors, when participants were asked directly which chatbot was easier to use, there was no difference between the conversational (118/224, 53% of participants) and the menu-driven (106/224, 47%) chatbot, with a Bayesian binomial test revealing moderate evidence in favor of the null (BF_01_=8.70). However, when asked which chatbot was most helpful, there was anecdotal evidence supporting a preference for the conversational (131/224, 58%) over menu-driven (93/224, 42%) chatbot (BF_10_=2.10).

Taken together, our findings suggest that although there were subtle differences in user-friendliness and overall trust between the menu-driven and conversational chatbot systems, these differences were not substantial enough to practically impact users’ perceptions of trust across chatbots. Trust levels for each chatbot were consistently just above the neutral midpoint, indicating a generally positive perception. When students were explicitly asked which chatbot they would prefer to use if they were to seek help, a majority appeared to favor the conversational chatbot (92/224, 41%), over a menu-driven chatbot (74/224, 33%) or choosing neither system (58/224, 26%). However, a Bayesian multinomial test run in the *JASP* statistics package [68] provided anecdotal evidence for the null (BF_01_=1.96), suggesting there was no statistical evidence for a preference regarding chatbot type.

Students provided 245 comments when indicating their preference for a chatbot. Slightly more commentary was provided regarding the conversational chatbot (n=88 in comparison to n=75 that specifically focused on the menu-driven chatbot), and of these 88 comments, a total of 66 were comments that supported the use and features of the conversational chatbot. These codes directly mentioned easy or easier use or interaction or understanding (n=12), being friendlier (n=1), more realistic (n=8), more relatable (n=2), more helpful (n=5), like a person/human (n=6). The codes also mentioned a greater flow of conversation (n=6) and the ability to frame the issue in their own words (n=14). Students also spoke about the conversational chatbot’s ability to tailor the conversation or interaction to their specific problem (n=5). Students spoke of the perception of the conversational chatbot taking the burden of the problem (n=4) and helping them navigate to meaningful resources (n=4). Of the 75 menu-driven chatbot comments, a total of 65 were in support of the use and features of the menu-driven chatbot. These codes directly mentioned easy or easier use or interaction or understanding (n=10), being efficient (n=5), more realistic (n=8), access to resources (n=11), more helpful (n=5), quick access to information (n=3), and helpful when interacting due to a menu, prompt, or list of options (n=8). Students found the menu-driven chatbot less invasive (n=5), found that the menu prompts helped frame the issue they were facing (n=6), and preferred the option of not having to type (n=5). Concepts of “trust” were directly mentioned (n=6); however, these were either in the context of not trusting AI, “AI doesn’t feel supportive or trustworthy,” or in defining what a trusted person would look like (“Trusted person would be patient”).

Students differed in terms of their prior experience with chatbots similar to the conversational one used in this study, with the majority of participants (134/224, 60%) reporting no previous interaction with a similar conversational chatbot, while 33% (73/224) had prior experience, and 7% (17/224) were uncertain about their past interactions. A user’s degree of prior experience with similar chatbots may adversely affect participants’ perceptions of the conversational chatbot regarding overall trust and user-friendliness. However, prior experience did not affect either overall trust (BF_01_=13.04) or perceptions of user-friendliness (BF_01_=9.35; Table 4 provides descriptive statistics).

**Table 4:** Mean ratings of overall trust and user-friendliness for the conversational chatbot, according to prior experience with conversational chatbots.

Measure	Response	Rating, mean (SD)
Overall trust	Yes	3.44 (0.82)
Overall trust	No	3.49 (0.75)
Overall trust	Unsure	3.52 (0.62)
User-friendliness	Yes	3.37 (1.07)
User-friendliness	No	3.47 (0.97)
User-friendliness	Unsure	3.65 (0.86)

Unsurprisingly, anonymity emerged as a significant concern among students, with 69% (155/224) expressing a preference for anonymous interactions with an online safety chatbot; only 10% (22/224) preferred nonanonymous interactions, while 21% (46/224) were undecided. These results confirm the importance of user privacy to adolescents in an e-safety context. When asked about personalizing a conversational chatbot, such as giving it a face or name, 39% (88/224) favored personalization, 34% (75/224) preferred a nonpersonalized chatbot, and 27% (61/224) were uncertain, demonstrating no significant preference for personalization of the chatbot. Thus, while some users may want a more engaging and relatable interaction with the chatbot, others do not perceive the need to add human-like characteristics in this context.

Students directly mentioning the conversational chatbot included some concerns or issues with the conversational chatbot (n=11). These comments were focused on unhelpful responses from the chatbot (output did not make sense from input given, n=5), causing the student to hesitate in interaction (due to needing to type out the issue, n=1), perceiving to not provide advice (n=1), causing greater distress (due to misunderstanding from the chatbot, n=1), or uncomfortable (n=1), hard (n=1), and artificial (n=1) to interact with. Student comments directly mentioning the menu-driven chatbot also included concerns or issues with the menu-driven chatbot (n=6). These comments focused on the menu-driven chatbot’s inability to handle or misunderstand student queries (n=3), the perception of the platform as more closed off (n=1), unhelpful (n=1), and limited in the range of topics it could offer advice on (n=1).

Of the remaining 82 comments (total comments n=245), students spoke about a negative view of turning to AI. Some students did not feel they could trust (n=3) or turn to AI (n=2), and some felt that using AI for help with online harms was inappropriate (n=8). Some students preferred to turn to people they trust (n=9) or look up the information themselves (n=5). Some students stated they did not prefer either chatbot (n=10). While others did not have a clear answer (n=2) or did not know which chatbot they preferred (n=10). However, some identified that both chatbots were appropriate/relevant resources (n=5). A few students expressed feeling confused (n=2) or frustrated (n=1) by the responses provided by either (or not specifically named) chatbots. The students identified the potential serious consequences of chatbots failing to provide correct or meaningful interaction during crises (n=2). Students directly spoke about improving the conversational chatbot (n=10), noting its limited conversation abilities and the need for trust in the interaction (n=6), as well as a more human-like approach to engage users (n=2). Additionally, students spoke about the need for the chatbot to provide advice, not just links (n=5), and felt that better options were already available (n=4). Thus, overall, students identified the importance of improving chatbots for more meaningful interactions and advice, highlighting the need for trust and a human-like approach.

### Summary of Key Findings

A substantial percentage of respondents (87/224, 39%) expressed a willingness to seek help outside their family and social circle when dealing with online harms, and approximately 60% (conversational chatbot: 141/224 and menu-driven chatbot: 142/224) of respondents engaged with our chatbot systems. When dealing with chatbot systems, our results highlight 2 related issues of importance. First, trust was strongly associated with willingness to click links provided by our chatbots. Perceptions of trust were generally similar across chatbot types and for male and female participants. Second, most respondents (155/224, 70%) expressed a preference for anonymity when dealing with chatbot systems. Thus, to be effective, and capitalize on adolescents’ willingness to seek external support, chatbot systems must be able to build and maintain trust with users, and it will be necessary to assure users of their continued anonymity.

## Discussion

### Overview

Adolescents’ vulnerability to online dangers, and the associated potential negative impacts on their mental well-being, highlight the urgent need to address cyber safety issues among young people. It is essential to provide young people easy access to trustworthy information and support. Chatbots represent a promising tool in addressing cyber safety concerns, offering convenient access to established and reputable support services, and helping link students with appropriate clinical support providers. We explored whether trust in chatbot systems was influenced by the design of the chatbot. Examining students’ perception of 2 chatbot systems, a menu-based and conversational chatbot, in terms of key trust-related factors could inform the development of an effective user-centered chatbot in the educational online safety context.

### Trust and Chatbot Efficacy

Individuals usually need to feel trust before following recommendations [11,17-19,21]. Thus, establishing and maintaining trust in an e-safety chatbot is vital to ensure adolescents are comfortable using the system and receptive to its suggestions for online safety resources. Comparing adolescents’ perceptions of trust for menu-driven and conversational chatbots allowed us to examine which interaction method and design approach better suited students’ preferences. As trust is multifaceted, we evaluated how key trust-related factors (ie, perceived accuracy of information, user-friendliness, appropriateness of the chatbots’ recommendations, and feelings of trustworthiness, security, and positivity toward the interaction) impacted overall trust across chatbot systems. Both chatbots were viewed more positively than negatively in terms of overall trust, indicating that they are both viable options for a trusted online safety chatbot. When students were directly asked which chatbot they would prefer for seeking help, there was some evidence for equivalence, suggesting no meaningful difference between the 2 options, despite a higher number of students endorsing the conversational chatbot over the alternative. Student comments supported the endorsement of the conversational chatbot, highlighting its “conversational nature” and “interactive” elements that simulated real-life conversations, “it was easy to communicate with and share my deepest and darkest thoughts” and “You are able to describe what you need help with in greater detail.” The perceived ability for the conversational chatbot to emulate person-to-person interaction facilitated some preference for this mode of delivery, “the conversational one was easier to understand and felt like i was talking to someone rather than clicking a menu.” However, there remains room for improvement based on the mean trust ratings.

### Help-Seeking Behavior

Over a third of participants were open to seeking help beyond their friends and family, indicating that many young people could benefit from support provided by an online safety chatbot. Further, there is a positive link between the intention to use tech tools, like chatbots, and their actual usage [44]. Thus, to assess users’ readiness to engage with the chatbot’s recommended resources, we included clickable help links to external support sites and asked users whether they clicked a link. The majority of students reported clicking a support link, with no difference between chatbot systems, indicating the interface type did not impact the likelihood of accessing recommendations for further assistance. Links were perceived by students as being helpful, with one student mentioning, “it provides useful links for kids/teens in a way that makes them feel more comfortable without seeking outside help.” However, another student suggested integrating the links into the conversation, “the links should be embedded into script rather than just being a plain link, it is not a big step but it definitely makes you more likely to click on a link.” There was some, albeit weak, evidence that males were less likely than females to click on help links, aligning with the well-documented gender disparity in help-seeking behavior [69]. Although participants in this study reported identifying as either male or female, it is also important to consider the perspectives of gender-diverse populations, as this at-risk group often faces extra challenges and unique concerns. Understanding specific gender-related issues that could hinder help-seeking is important for developing an inclusive online safety chatbot that caters to all young people. Unfortunately, gender-diverse populations were under-represented in our sample.

Trust was an important factor in user behavior within chatbot interactions, as the level of trust students had in the chatbot was related to the likelihood of clicking a link (engaging with a linked support service) within the chatbot interaction. Additionally, we found choosing to click on a link was related to a higher sense of positivity regarding that choice, as opposed to opting not to press a link. The underlying mechanism here can be interpreted in different ways. One possibility is that a feeling of positivity arises from the decision itself, suggesting an associated sense of satisfaction in taking a proactive step toward seeking help. Alternatively, it could reflect a preexisting state of positivity or readiness to engage with the provided resources, influencing the decision to press the help link. Further examination of these mechanisms might provide greater understanding of users’ decisions to seek help and their interactions with a chatbot, particularly concerning the inclusion of links to external support resources and how they perceive these resources. This may inform strategies aimed at improving the chances of adolescents accessing online support when needed.

### Design Implications: Empathy, Personalization, and Anonymity

To explore students’ interest in an online safety chatbot, we used a simple conversational chatbot, which operates with predefined outputs that are matched to conversational intents. Thus, although the current results serve as a useful proof of concept, the data from this study also highlight potential areas of improvement, particularly in terms of increasing cognitive trust (through accuracy and user-friendliness) and affective trust (through empathetic responses and personalization). The simple conversational chatbot appeared to struggle at times when handling students’ written input. One student expressed this frustration: “Sometimes I found it hard to specifically say the situation and get the bot to understand.” An inability to handle requests effectively may have lowered trust in the chatbot’s competence and would seem like a substantial issue in applied settings. As another student noted, “it just kept saying “please re-phrase that” or “thats [sic] not relevant to the issue” when it was.” These issues will need to be addressed to improve user experience and effective delivery of online safety support. User-friendliness could be improved by training the underlying machine learning model on training data from adolescents to better establish the user intent. The current chatbot was primarily trained on adults, with input from only a handful of younger adults, and based on comments made during the focus groups conducted during the co-design process. Additional sampling of several hundred, or thousand, students could create a much larger training dataset to produce a more robust model of user intent (the ethics approval for this study precluded the evaluation data being used as a training dataset for the model). While a menu-driven chatbot’s simple interface with clear options makes navigation easy, predictable, and reliable, its standardized interactions may reduce emotional connection with users. In contrast, a conversational chatbot can be more emotionally engaging due to its capacity for natural language and empathetic responses. The simple conversational chatbot used in this exploratory study was focused on delivering support links and had limited natural interaction and empathic capabilities. Based on feedback from students (ie, the intended users of the chatbot), it seems that there was a desire for a more empathetic and personalized chatbot. For example, one student stated:

The understanding of certain kid/teens phrases or slang… more of a response aside from a brief description and a link, something like what snapchat AI would respond with more care and emotion.

Future research should explore enhancing empathetic interactions, such as using warmer language and including more reassuring phrases, as prior studies indicate the potential benefits of a strengthening the user connection with the chatbot [2,70-74]. By incorporating these improvements into future designs, we may create an engaging and informative system, leading to a positive user experience and effective delivery of online safety support.

Personalization features, such as giving a chatbot a face or name, have the potential to enhance user engagement and satisfaction by creating a more personalized and human-like experience [30,71]. However, our current findings indicate that while some users want a more engaging and relatable interaction with the chatbot, others do not see the need for human-like characteristics. One student stated, “I think that if the chatbot was personalised it would help develop more of a connection with those seeking support and as though they aren't talking to a robot” while others preferred a nonpersonalized chatbot, explaining:

Having a non personalised Chatbot makes the information feel more trustworthy and reliable…” and “Giving the chat bot a face would also make it harder to communicate your problems, I believe it should be similar to a confessional booth.

This was surprising, as studies have found including a human-like visual cue in a chatbot can improve trust and reduce the psychological distance between the user and the chatbot [3,33,71,75]. However, the opposition, or hesitation, to personalization among some participants may stem from concerns related to privacy—known as the “valley effect,” where users become increasingly concerned about privacy as technology becomes more human-like in its interactions [76,77]. Thus, if personalized features were to be incorporated in future designs, there may be a need to clearly address concerns related to privacy to ensure users feel comfortable and trusting in their interactions with the chatbot.

Anonymity was a significant concern among the students, with an overwhelming preference that an online safety chatbot should be anonymous. When users feel confident that their conversations are confidential, they are more likely to engage openly and honestly with the chatbot. One student noted that “Having the chatbot clearly anonymous will encourage openness” while another explained, “Anonymity is essential for someone to share their true, unfiltered opinions on the internet, to a 'secure' bot or to another person.” Many users may be dealing with sensitive issues, such as online harassment, image-based abuse, substance abuse, or mental health challenges. They may feel a sense of shame or embarrassment if this information becomes known. As one student noted, “Having this privacy could encouraged [sic] those that may be embarrassed by their situation or not willing to share when their name is attached to it.” Another mentioned, “…it provides the person in need with a safe outlet whilst also giving them a reliable source that they dont [sic] have to be ashamed about.” The sharing of personal information can facilitate better support and guidance tailored to their individual needs. A chatbot service can provide a safe, confidential space for adolescents to share their concerns to help them navigate their problems. On a related note, it will be vital that data security measures ensure the privacy and confidentiality of any data supplied by users accessing a chatbot system like this in applied settings. Any misuse of data provided by users would not only be extremely unethical, but would rightfully damage users’ trust in the chatbot system and consequently negate its efficacy.

### Limitations

Given that this is a proof-of-concept study, there are limitations to the generalizability of the results obtained. Some of these limitations relate to the number and nature of vignettes explored and to the nature of the sample tested. Obviously, a chatbot may be accessed as a source of support by individuals experiencing a wide range of personal circumstances, and our stimulus and participant sampling does not provide a comprehensive representation of population of interest (eg, our data do not speak to how user characteristics like ethnicity, gender diversity, or sexual orientation affect the actual or perceived utility of the chatbot). Moreover, although our data are promising and serve as a proof-of-concept, promising findings in controlled, simulated testing environments do not guarantee efficacy in field settings. Thus, although our data provide initial support for the utility of chatbots in supporting the needs of adolescents at risk of online harms, further work is required to establish the generalizability of these findings. Further, there are aspects of trust and of chatbot technology that require additional attention. A trust-related factor we did not examine, but that requires attention, is that trust in technology systems can be influenced by the credibility of the organization involved, and their knowledge base (ie, that provides advice and guidance) [78]. In the context of health-related services, trust in the health information provider is crucial in increasing compliance with professional advice [79,80]. If the associated organization lacks credibility, people are less likely to follow recommendations [11,21]. However, as adolescents have identified schools and reputable online safety websites as their preferred sources for accessing online safety information [45], a school- or community-based system has the potential to gain students’ trust. Thus, in the context of an online safety chatbot, its association with reputable institutions like schools and government-funded organizations dedicated to online safety (eg, Butterfly Foundation, Headspace) may improve access to quality support for cyber safety issues.

Another issue is that chatbot technology continues to evolve, as large language models (LLMs) provide an opportunity to create more lifelike and realistic dialogue. These LLMs are advanced AI models trained on vast amounts of text data, which could be selected to be appropriate and realistic for an adolescent audience. They are capable of ingesting and then generating human-like text across a wide range of topics. While LLMs are not specifically chatbots, they can be used as the underlying technology for conversational agents. They excel at generating coherent and contextually relevant responses to user input, even in complex conversational scenarios. LLMs are highly versatile and can be fine-tuned for specific tasks, including chatbot applications, content generation, and language translation. Such chatbots, as with the curated set of resources used in the chatbot in this study, will require maintenance, as LLMs are only aware of resources and supports contained within their training datasets. Currency and relevancy of support provided will be an ongoing challenge for each chatbot architecture.

### Conclusion

Young people understand the value of obtaining more information about online risks, not only to deal with their own negative experiences but also to support others in similar situations [45]. This information also plays a role in promoting responsible online behavior, as teens might unknowingly cause harm due to their limited understanding of legalities, consent, and privacy. An online safety chatbot has the potential to serve as a valuable supplementary cyber safety resource alongside school support systems, such as pastoral care or school psychologists, with the aim to increase accessibility for adolescents navigating online safety challenges. Integrating a chatbot into schools’ digital libraries, resources, or a student portal, can promote a collaborative approach to e-safety education and support. Online safety chatbots guided by a framework of trustworthiness can support users in navigating online risks and accessing valuable resources. In designing and deploying an online safety chatbot, it will be essential to build trust to ensure users feel emotionally connected and comfortable with its use in navigating online risks. This technology-driven approach may be particularly helpful for those who prefer digital interactions or feel apprehensive seeking help directly from others. An online safety chatbot can offer a confidential and easily accessible space where adolescents can seek guidance and access information from reputable organizations dedicated to young people and online safety.

## Abbreviations

AI: artificial intelligence
BF: Bayes factor
CrI: credible interval
LLM: large language model
NHST: null hypothesis significance testing
